# Human-Wildlife Conflicts in Nepal: Patterns of Human Fatalities and Injuries Caused by Large Mammals

**DOI:** 10.1371/journal.pone.0161717

**Published:** 2016-09-09

**Authors:** Krishna Prasad Acharya, Prakash Kumar Paudel, Prem Raj Neupane, Michael Köhl

**Affiliations:** 1 Department of National Parks and Wildlife Conservation, Government of Nepal, Kathmandu, Nepal; 2 Nepal Academy of Science and Technology, PO Box 3323, Khumaltar, Lalitpur, Nepal; 3 University of Hamburg, World Forestry, Leuschnerstr 91, D-21031, Hamburg, Germany; 4 Friends of Nature, Kathmandu, Nepal; Sichuan University, CHINA

## Abstract

Injury and death from wildlife attacks often result in people feeling violent resentment and hostility against the wildlife involved and, therefore, may undermine public support for conservation. Although Nepal, with rich biodiversity, is doing well in its conservation efforts, human-wildlife conflicts have been a major challenge in recent years. The lack of detailed information on the spatial and temporal patterns of human-wildlife conflicts at the national level impedes the development of effective conflict mitigation plans. We examined patterns of human injury and death caused by large mammals using data from attack events and their spatiotemporal dimensions collected from a national survey of data available in Nepal over five years (2010–2014). Data were analyzed using logistic regression and chi-square or Fisher's exact tests. The results show that Asiatic elephants and common leopards are most commonly involved in attacks on people in terms of attack frequency and fatalities. Although one-horned rhinoceros and bears had a higher frequency of attacks than Bengal tigers, tigers caused more fatalities than each of these two species. Attacks by elephants peaked in winter and most frequently occurred outside protected areas in human settlements. Leopard attacks occurred almost entirely outside protected areas, and a significantly greater number of attacks occurred in human settlements. Attacks by one-horned rhinoceros and tigers were higher in the winter, mainly in forests inside protected areas; similarly, attacks by bears occurred mostly within protected areas. We found that human settlements are increasingly becoming conflict hotspots, with burgeoning incidents involving elephants and leopards. We conclude that species-specific conservation strategies are urgently needed, particularly for leopards and elephants. The implications of our findings for minimizing conflicts and conserving these imperiled species are discussed.

## Introduction

Conflicts between people and wildlife have been widely recognized as one of the most challenging issues for wildlife conservation worldwide [1,2]. Although problems have been well known for many years, the increase in conflicts, particularly in regions with high biodiversity, suggests that improved strategies are urgently needed to promote the co-existence of wild animals and people [2,3]. The continuous increase in the human population results in competition between people and wildlife for shared but limited resources, which manifest as various types of conflict, such as crop-raiding, livestock predation, property damage, human death and injury, and the retaliatory killing of wildlife [4,5]. Conflicts become extremely controversial when people are attacked by species that are endangered and legally protected. First, attacks by wildlife are life-threatening and thus are not acceptable to society, so people often retaliate by killing the animals involved in the conflict [6]. Second, large mammals are generally involved in the conflicts, and most of these species are threatened with extinction, so the retaliatory killings of threatened mammals further increases their extinction risk [7,8]. Third, the penalties for illegally killing endangered animals may further escalate hostile attitudes towards conservation efforts [9].

Several measures, ranging from the distribution of compensation and the promotion of wildlife deterrents to support the livelihoods of people, have been implemented to foster the co-existence of humans and wildlife [2,3,5,10]. However, the efficacy of such measures is largely uncertain due to the absence of information about the patterns of conflicts across various landscapes. Although human-wildlife conflicts have been extensively studied at local levels [11–13] and to some extent in Nepal [14–16], none of these studies report patterns of human fatalities and injuries caused by wild animals at the national level, with some exceptions in Africa [17,18].

**Fig 1:**
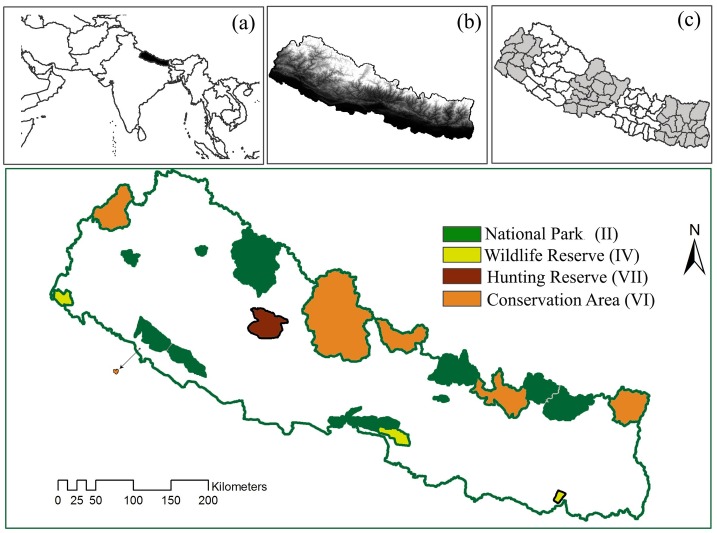
Map of protected areas in Nepal. Figures in parentheses indicate IUCN (World Conservation Union) protected area categories. In inset: (a) location of Nepal (dark color), (b) altitudinal gradient in Nepal (the lighter colors indicates higher altitudes), and (c) districts of Nepal. Five clusters of districts (indicated by shades of colors or white) indicate jurisdictions of the Regional Forest Directorate (RFD).

Nepal, a central Himalayan country, has an exceptionally high level of biodiversity, partly because of the large variation in altitude (70–8,848 m) that occurs over short horizontal distances (~200 km) (Fig 1). The country has a disproportionately high diversity of flowering plants (~2% of the global number of species), mammals (8%) and birds (8.6%) in comparison with its proportion of global landmass (<0.01%) [19]. Maintaining biodiversity in this country is ranked as a very high global conservation priority, as demonstrated by efforts to maintain endemic bird areas [20] and the inclusion of areas of the country in the Global 200 ecoregions identified by the WWF [21]. Nepal has 23.24% of its land mass in protected areas (PAs) (Fig 1). Outside the PAs, approximately 29% of the forestland is managed under community forestry practices, where local communities play a significant role in forest management and decision-making about land use. Conservation challenges in such areas are complex and are mostly associated with the socio-cultural status of the people living there [19,22].

Protected areas in Nepal are disproportionately located at higher altitudes [23]. Consequently, the fauna of the lowland regions, especially large mammals, are not adequately protected, and most of them live in human-dominated forest landscapes [24]. The country has an unusually high proportion of globally threatened species of mammals in comparison to its area [8]. Nepal is a predominately agricultural country, with forests providing many life-supporting ecological goods and services. For example, firewood and fodder make up nearly 75% of the energy supply and 37% of the livestock feed, respectively, used by the country, and these are mostly harvested from forests [25]. A close link between society and the natural environment and their close physical proximity are a major cause of human-wildlife conflicts. Various reports suggest that there is an increasing incidence of human causalities and injuries due to wildlife interactions, even in areas with no previously reported incidents [12,14,16,26]. Therefore, measures based on sound analyses of the spatial and temporal patterns of human casualties and injuries are needed to reduce the frequency of these conflicts.

In Nepal, people are attacked by large mammal species such as tigers, common leopards, rhinoceros, elephants and bears, but there is little discussion about the patterns of fatalities and injuries caused by wildlife or their underlying temporal dynamics [27]. Such information could provide essential guidance for establishing future conservation and research priorities in Nepal [19]. In this paper, we analyze data on human-wildlife conflicts collected over a five-year period (January 2010-December 2014) via a nation-wide survey of district forest offices and PA offices (districts and PAs are shown in Fig 1). The aims of this study were to (1) explore the temporal patterns (year, season and month) of wild animal attacks on people for different species, (2) determine the locations most vulnerable to attacks (e.g., home, forest and farmland),(3) identify conflict hotspots in Nepal, and (4) provide recommendations to support future conservation planning in Nepal.

## Materials and Methods

### Data assessment

We assessed data on human fatalities and injuries obtained from the Regional Forest Directorates (RFDs) and the Department of National Parks and Wildlife Conservation (DNPWC). The Ministry of Forests and Soil Conservation (MoFSC) implemented guidelines for relief payments for wildlife-related losses in 2006 (with an amendment in 2015). The guidelines provide a systematic procedure for providing financial support to victims or their dependents for various types of losses caused by wildlife: (1) loss of human life or injury, (2) loss of livestock, (3) loss of crops and stored food-grain, and (4) damage to houses and farm buildings. To avoid unjustified claims, the guideline stipulates a rigorous verification protocol that includes plausibility checks and objective evidence. According to the guidelines, the RFD is the entity responsible for the approval and disbursement of financial support to victims. (S1 File). In addition, we made telephone calls to district forest offices and PA offices to verify data and assess if there were any unreported and/or undocumented cases. We found that most relief claims were for human fatalities and injuries, while claims for crop and livestock loss were not common.

We prepared a database with 463 conflict cases involving death or injury of people caused by wildlife over a five-year period (2010–2014). The data indicate that bear, gaur (*Bos gaurus*), Asiatic elephant (*Elephas maximus*), common leopard (*Panthera pardus*), one-horned rhinoceros (*Rhinoceros unicornis*), Bengal tiger (*Panthera tigris tigris)*, wild water buffalo (*Bubalus arnee*) and wild boar (*Sus scrofa*) attacks on people all occurred during this period. For leopards, all attacks were by common leopards. Attacks by snow leopards (*Uncia uncia)* are very unlikely as they are not found below 3000 m [28], and our database suggests that leopard attacks occurred only in the mid-hills and the lowlands.

For each conflict event, we attempted to document the following data: (1) type of conflict (death or injury); (2) species involved; (3) time of incident (year, month, and season) (winter: December-February; spring: March-May; summer: June-August; autumn: September-November); (4) location of conflict (forest, farmland, or home); and (5) whether the conflict was inside or outside existing PA boundaries. The ‘home’ conflict location covers the homestead, including the house, livestock sheds, other structures, gardens and nearby vegetable plots, while ‘farmland’ includes land used for agricultural production (Table 1).

**Table 1:** Patterns of human death and injurydue to large-mammal attacks (mean and standard deviation) in the period from 2010–2014. Statistics for the ‘other’ category are not shown. Average (with ± SD).

Wildlife	Contribution[%]	Average number of attacks per year^a^	Average number of fatalities per year^a^	Average number of attacks per season^b^	Average number of fatalities per season^b^
Elephant	30	27.4 ±7.7	18 ±4.6	34.2 ±16.5	22.5 ±11.7
Leopard	21	19.4 ±11.6	8±5.4	24.2±3.8	10±6.6
Rhinoceros	18	17 ±4.3	3±1.2	21.2±16	3.7±3.5
Bear	12	11 ±4.3	1±1.2	13.7±2.6	1.2±1.2
Tiger	10	8.8±5.4	4.8±3.3	11±4.8	6±1.4

^a^observation period = 5 years

^b^number of seasons per year = 4

### Data analysis

We classified each incident as either a fatality or injury, coded as 1 or0, respectively. Some species, such as gaur, wild water buffalo and wild boar were grouped in an “other” category as only a few cases involving these species were reported in certain seasons. We computed the kill prevalence and incident prevalence for each species as the percentage of the total number of fatal events and the percentage of the total number of incidents, respectively. Chi-square tests of independence or, in cases where there were a small number of observations, Fisher’s exact tests were applied to compare the frequency of attacks (fatalities and injuries) by each wildlife species in relation to time (year, season, month), location (forest, farmland and home) and whether they were inside a PA boundary. We used a logistic regression (generalized linear model with a binomial error distribution and logit as the link function) for modeling season, wildlife category, and location (home, farmland and forest) as predictors of increased probabilities of fatalities and injuries in cases of attacks. The R statistical environment (R Development Core Team, 2015) was used for all analyses.

## Results

### Overall conflict pattern

**Fig 2:**
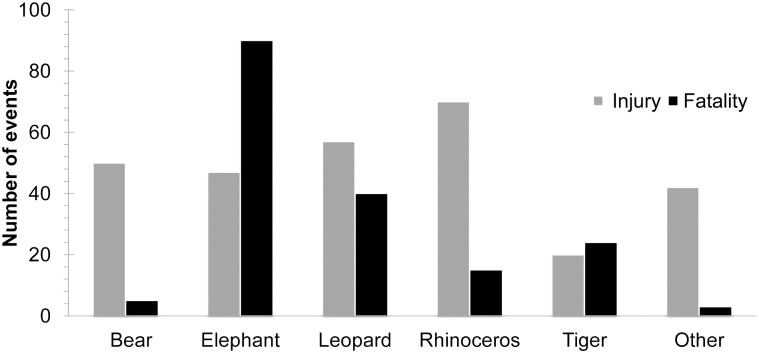
Frequency of attacks (fatalities and injuries) by bears, elephants, leopards, rhinoceros, tigers and others (gaur, water buffalo and wild boar) from 2010–2014.

Our data show that wildlife encounters with people resulting in death or injury in the five-year period from 2010 to 2014 involved the following animals: elephants (30%), leopards (21%), rhinoceros (18%), bears (12%), and tigers (10%) (Table 1, S2 File). On average, 7.7 attacks, including 2.9 fatalities, were reported per month (Table 1). The differences between the frequencies of fatalities and injuries were significant among wildlife species (X^2^ = 103.1, df = 5, P<0.001) (Fig 2). Among the species analyzed, three were significantly associated with human deaths: elephants (kill rate = 0.66, P<0.001), leopards (kill rate = 0.41, P = 0.002), and tigers (kill rate = 0.55, P = 0.005) (Fig 2).

**Fig 3:**
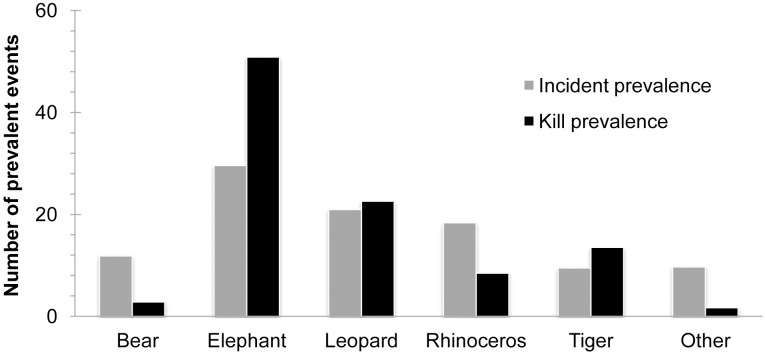
Incident prevalence and kill prevalence forbears, elephants, leopards, rhinoceros, tigers and others (gaur, water buffalo and wild boar) during the period from 2010–2014.

Overall, there was a significant difference between the incident prevalence and kill prevalence (X^2^ = 21.25, df = 5, P = 0.0001), and for elephants and tigers, the kill prevalence exceeded the incident prevalence (Fig 3).

### Temporal pattern of human injuries and fatalities

**Fig 4:**
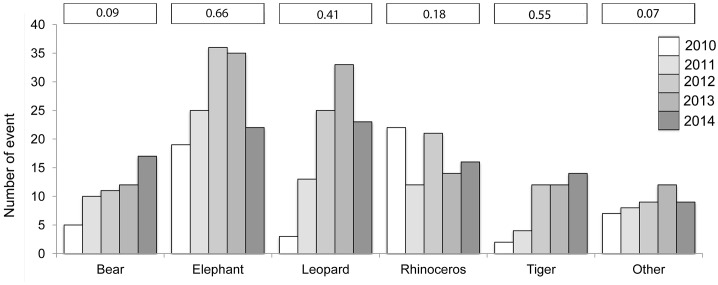
Frequency of attacks by bears, elephants, leopards, rhinoceros, tigers and others over a five-year period (2010–2014) by year. Numbers in a vertical line along the x-axis are the average kill rates of each wildlife species.

We detected an increased frequency of wildlife attacks from2010 to2014 for bears (R^2^ = 0.91), leopards (R^2^ = 0.67), others (R^2^ = 0.45) and tigers (R^2^ = 0.87). For elephants, the trend was less pronounced (R^2^ = 0.11), and it was negative for rhinoceros (R^2^ = 0.13) (Fig 4). There were statistically significant differences among wildlife species in terms of total attacks (X^2^ = 38.7, df = 20, P = 0.007) and kill rates (X^2^ = 153.43, df = 20, P < 0.001).

**Fig 5:**
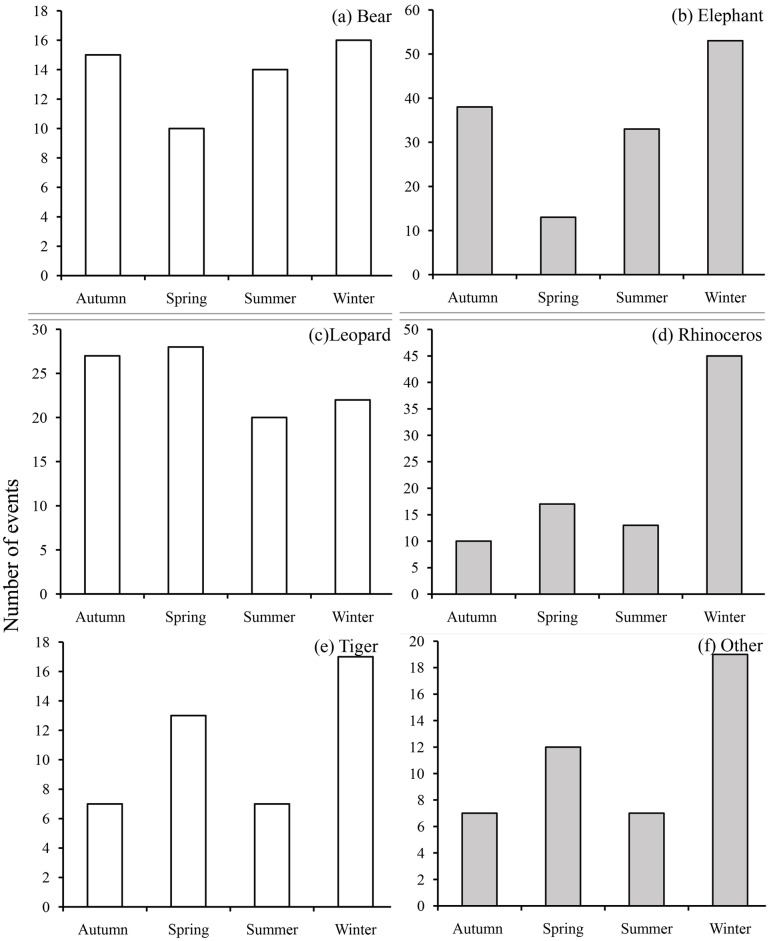
Frequency of attacks (both fatal and those causing injury) by bears, elephants, leopards, rhinoceros, tigers and others over the five-year period by season in Nepal:(a) autumn, (b) spring, (c) summer, (d) winter. Grey-filled bars indicate a statistically significant difference in the seasonal attack pattern.

We detected significant seasonal variations among the wildlife species when we analyzed the data for the frequency of attacks (X^2^ = 40.27, df = 15, P < 0.001), frequency of deaths (Fisher's exact test, P = 0.01), and kill rates (Fisher's exact test, P < 0.001) over the five-year period. Attack frequencies differed significantly among the seasons for elephants (X^2^ = 23.905, df = 3, P<0.001), rhinoceros (X^2^ = 36.553, df = 3, P<0.001) and others (X^2^ = 8.6, df = 3, P = 0.03), with a higher frequency of attacks consistently occurring in winter. There were no significant seasonal variations in the frequency of attacks by tigers (P = 0.08), bears (P = 0.68) or leopards (P = 0.60) (Fig 5). However, the frequency of fatalities caused by leopards varied significantly with season (X^2^ = 13.4, df = 3, P = 0.003), with a higher frequency of kills observed in autumn.

**Fig 6:**
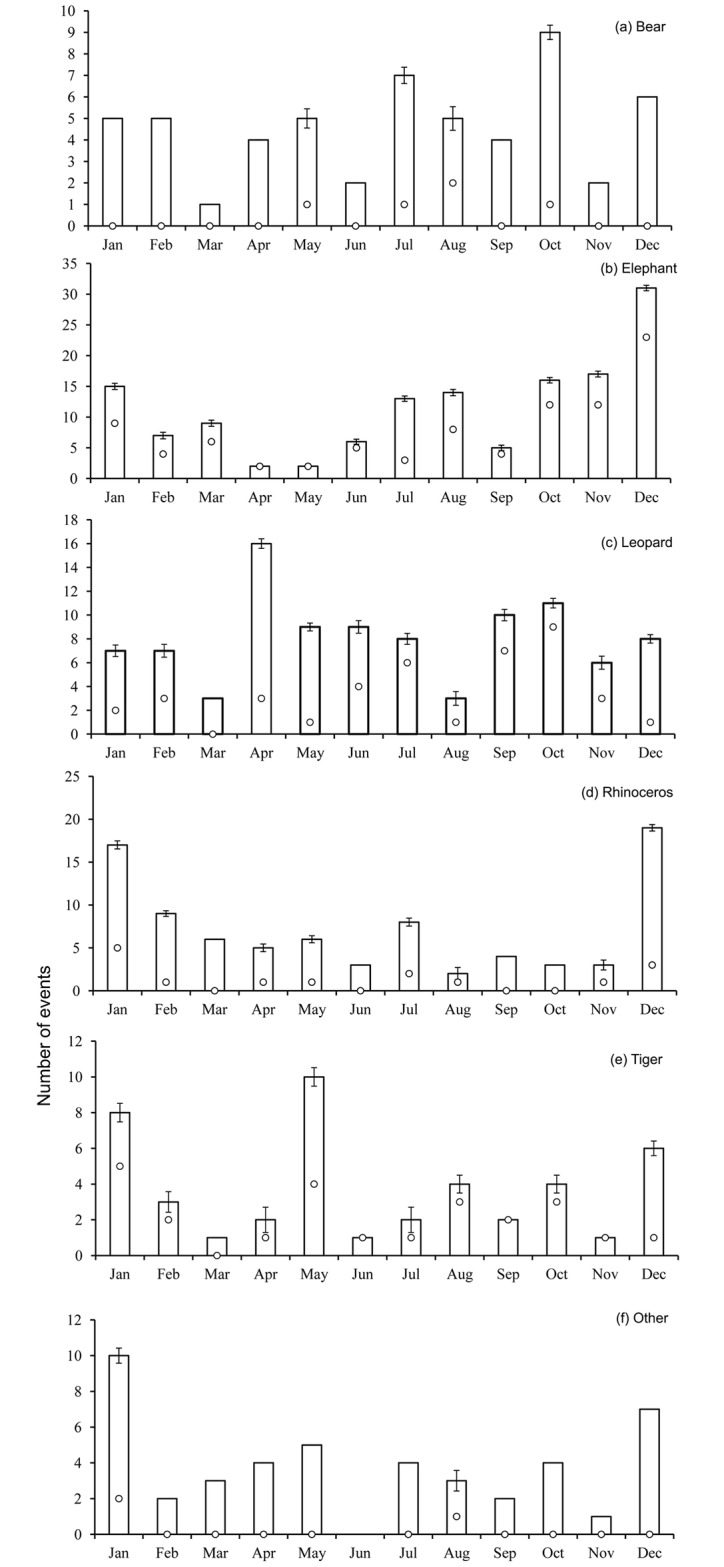
Frequency of attacks (fatal and those causing injury) by month over a five-year period (2010–2014) by (a) bears, (b) elephants, (c) leopards, (d) rhinoceros, (e) tigers, and (f) others. White circles indicate kill ratios. Error bars indicate the standard deviation of fatal events for the corresponding wildlife species and month.

Attacks by wildlife differed significantly across the months (Fisher’s exact test, P<0.001). Attacks by elephants were more frequent in December and less frequent in April and May (Fig 6b). Leopard attacks occurred mostly in April (Fig 6c), while tiger attacks occurred most often in January and May (Fig 6e). Rhinoceros in particular showed a distinct pattern, attacking humans more often in December and January (Fig 6d). The incidence of attacks by bears and others were not consistent throughout the year (Fig 6f). Generally, fatalities were significantly associated with month (P = 0.02), showing a higher frequency in September (P = 0.04) and October (P = 0.02).

### Spatial pattern of the occurrence of human injuries and fatalities

**Fig 7:**
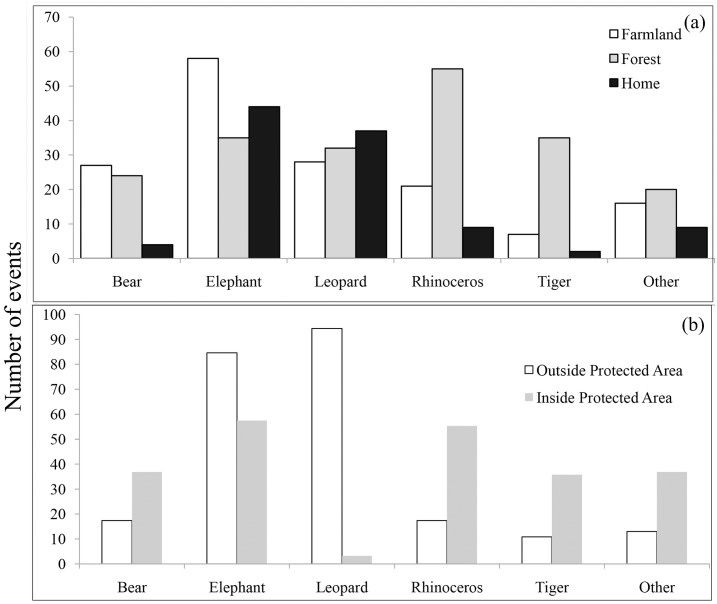
Spatial pattern of the occurrence of human injury and death caused by large mammal attacks in Nepal (a) in farmlands, forests, and homes, and (b) inside and outside protected areas.

Generally, attacks by wildlife were significantly associated with the location in which they occurred: home, farmland and forest (Fisher’s exact test, P<0.01). We detected significantly different frequencies of attacks among the locations for elephants (X^2^ = 5.88, df = 2, P = 0.05), tigers(X^2^ = 43.13, df = 2, P <0.001) and rhinoceros (X^2^ = 40.18, df = 2, P<0.001). Attacks by elephants occurred more often in farmlands, followed by attacks at homes and in forests. Attack patterns of rhinoceros and tigers were consistently similar (Fisher’s exact test, P = 0.22); they attacked more often in forests, followed by attacks in farmlands and homes. Bears and others showed a statistically consistent pattern (Fisher’s exact test, P = 0.13), attacking mostly in farmlands, followed by attacks in forests and homes (Fig 7a).

The logistic regression analyses demonstrated a significant influence of location (P<0.001), season (P<0.001) and species(P<0.001) on the likelihood of death resulting from an attack.(Table 2).The odds that a person would be killed in an attack were highest for elephants, followed by those for tigers (Table 2).

**Table 2:** Results of the logistic regression analyses showing the effect of location, season and species on human fatalities.

	Estimate	Std. Error	z value	Pr(>|z|)	Odds ratio
(Intercept)	-1.96	0.51	-3.79	0.0001	
Location					
Forest	0.19	0.27	0.70	0.480	1.21
Home	0.96	0.30	3.17	0.001	2.63
Season					
Spring	-1.19	0.35	-3.34	<0.001	0.30
Summer	-0.55	0.33	-1.65	0.097	0.57
Winter	-0.71	0.30	-2.37	0.017	0.48
Wildlife					
Elephant	2.82	0.51	5.51	<0.001	16.85
Leopard	1.75	0.52	3.32	<0.001	5.80
Other	-0.38	0.77	-0.49	0.621	0.68
Rhinoceros	0.83	0.56	1.47	0.139	2.29
Tiger	2.66	0.57	4.62	<0.001	14.39

There were significant differences between the frequencies of attacks (or events) by wildlife inside and outside PAs (X^2^ = 130.56, df = 5, P <0.001). Bears, rhinoceros, tigers, and others consistently attacked people inside Pas (X^2^ = 3.3, df = 3, P = 0.34) (Fig 7b). However, elephants and leopards attacked people more often outside PAs, although there was a significantly different attack pattern between them (Fisher’s exact test, P<0.001) (Fig 7b).

## Discussion

Understanding patterns of human-wildlife conflict and identifying the underlying causes are an important component of conservation biology. Our results shed light on the spatiotemporal patterns of human death and injury caused by large mammals at the national level and provide insight into future conservation needs. Elephants, leopards and rhinoceros were the top three conflict species in terms of total attacks, followed by bears and then tigers. For the kill/injury ratio, elephants ranked the highest, followed by tigers, leopards and then rhinoceros. Both the incident prevalence and kill prevalence were the highest for elephants, followed by those for leopards, rhinoceros and tigers. Thus, our results suggest that human-elephant and human-leopard conflicts are the most serious human-wildlife conflict challenges in Nepal. Furthermore, the preponderance of attacks occurred in human-dominated landscapes, which indicates the need for conservation management outside PAs.

Previous studies on human-elephant conflicts suggest that elephant attacks are common wherever elephants and people occupy the same space [16,29–32]. Elephants are the largest terrestrial mammal, and they roams vast areas while foraging for large quantities of food [33,34]. However, elephant habitats have been encroached upon to support the growing human population, resulting in the severe fragmentation of elephant populations and little to no interchange between them [34]. In Nepal, elephant populations are disjointed and confined to four small geographic clusters that were formerly part of an uninterrupted forest landscape that extended throughout southern Nepal and the adjoining region of northern India [35]. The pronounced occurrence of human-elephant conflicts may be attributed to (a) the loss of forests along seasonal migratory routes [33,34,36], (b) the shrinkage of available forested areas [31,37] and (c) direct contact with human populations who are dependent on subsistence agriculture [11]. All of these factors are consistent with our findings, especially in eastern Nepal. This region of Nepal has historically been part of a seasonal migration route between Meghalaya in India and central Nepal [19]. Although a large swath of forest that previously connected with India and spread across the Siwalik foothills was destroyed for human settlements, elephants continue to use the same routes, resulting in their presence in human dominated landscapes. The high frequency of elephant-human conflicts in farmlands and homes in the dry season (December) (Fig 5c) is associated with the crop harvesting months. This finding is similar to those of other studies in Nepal and India [16,32,38]. In December, natural food sources are very limited in the forest, so paddy crops (e.g., rice) that are ready for harvest become a target for hungry elephants. Parker and Osborn [39] noted that the cultivation of unpalatable cash crops (e.g., *Capsicum annuum*) on private land has been shown to be effective in reducing human-elephant conflicts in Zimbabwe, and this may be a solution for Nepal. However, a mitigation plan focusing on the ecological needs of elephants is of prime importance. Forests along elephant migration routes are already very small and fragmented, and future development plans (e.g., roads, railways, and airports) will further disturb these routes. Hence, it is critical that a strategic environmental assessment is conducted in light of the complex infrastructure development planned in this region [40].

Human-felid conflicts have been recognized as one of the major impediments to the future conservation of some of these most endangered species [11,15]. Our results show a surprisingly distinct pattern of attacks by leopards and tigers. Leopards had the second highest incident frequency in terms of total attacks on people and fatalities of the wildlife species analyzed, and leopard attacks peaked in April, the driest time of the year. We argue that common leopards have made a comeback in their former habitats in Nepal’s mid-hill mountains after the successful launch of the community forestry program. The program, administered by local residents at the community level, aims to meet local demands for fodder, firewood and timber; the biodiversity gains of community forestry are an unintended side effect [22,41]. Prey populations in community forests are likely to fluctuate due to shortages of food and water sources, especially in dry months [22,28,41]. Livestock are easy preys and their sheds are often poorly protected against leopard attacks. Therefore, people get attacked when leopards, particularly starving ones, resort to livestock depredation. The mid-hill mountain forests are generally not part of the PA system (this zone is very under-represented in Nepal’s PA network), and most forest patches are close to human settlements [23]. Studies have shown that leopards can live in human-modified landscapes [42,43], and the extent of human-leopard conflicts is associated with the depletion of nature prey populations, the scarcity of water and livestock herding and guarding practices [44]. Therefore, effective conflict mitigation strategies should include the conservation of leopards’ natural prey species in community forests (e.g., ban on wildlife hunting and habitat conservation and management) and the adoption of other measures (e.g., safe livestock enclosures, especially at night, and the herding of livestock outside of forests).

Tigers had a low attack prevalence compared with the four other major conflict species, and our findings suggest that attacks by tigers often occur in forested areas. Therefore, human disturbances in forests are the main reason for human-tiger conflicts. Similar to our results, Treves et al.[13] and Gurung et al. [14] reported that humans invading forests (e.g., pastoralists and fodder/firewood collectors) were often killed by tigers. Gurung et al. [14] found no seasonal pattern of attacks in Chitwan National Park, but attacks were spatially concentrated within the park boundaries, which is similar to our findings. Similar findings were also reported in Sumatra, where human-tiger conflicts are common in intermediate disturbance areas, such as multiple-use forests, where tigers and people coexist[45]. Carter et al.[46] found that tigers coexisted with people in disturbance areas by becoming nocturnal. Such findings suggest that tigers may be able to coexist with people, but it is reasonable to expect that human-tiger conflicts will increase in the future in Nepal for several reasons. Livestock constitute a large proportion (1–12%) of tigers’ diets [47–49]. The availability of wild prey is therefore critical in determining the level of human-tiger conflict. Although core tiger habitats have not been expanded in Nepal, restoration campaigns driven by the landscape conservation program in the Terai Arc Landscape [50] have enlarged the areas of multiple-use forests, many of which are managed by local communities. Such multiple-use forests may became conflict hotspots, as Gurung et al.[14] documented in the buffer zone of Chitwan National Park. Therefore, establishing zones of core tiger habitat outside PAs, with a particular emphasis on maintaining viable prey populations, is critical for minimizing human-tiger conflicts. This is also important for achieving Nepal’s commitment to the St. Petersburg Declaration, in whichthe government of Nepal committed to doubling its tiger population by 2025. This commitment is viewed differently by different experts; some find the targets of this plan highly ambitious [51], while others strongly support it [52]. We emphasize that improved habitat quality (e.g., increased prey populations and a reduced human footprint) is a pre-requisite for minimizing human-tiger conflicts and for gaining the support of communities for tiger conservation.

Rhinoceros occur in three locations in Nepal (Chitwan National Park, Bardia National Park, and Suklaphanta Wildlife Reserve); the latter two contain small, reintroduced populations. Rhinoceros were the species with the third highest prevalence of human-wildlife conflict at the national level. They attacked people primarily in the dry season (winter), and a large number of attacks took place in forests and farmlands. This was probably because of the geographical and temporal overlap that occurs between rhinoceros and people. Rhinoceros are active during the early morning [53] and wander into farmlands for opportunistic browsing, especially in the winter season when the quality and quantity of forage in forests are low [54]. Firewood and fodder collection, however, are major off-farm activities in the winter, and they take place in the early morning because of the short winter days. Our findings are in accordance with findings from Jnawali [55], who reported a high frequency of conflicts in farmlands and the adjoining forests. Our data suggest that there has been a decline in attacks by rhinoceros in recent years. This might be due to the increase in the use of electrified fences that separate rhinoceros populations from farmlands and settlements. Concurrently, tallgrass floodplain habitats and forage grass (e.g., *Saccharum spontaneum*), which are critical for rhinoceros [56], have been rapidly declining due to the succession of grasslands to woodlands (pers. observation) and the invasion of exotic plants such as *Mikania micrantha* [57]. Thus, habitat management within PAs needs to be urgently carried out to keep rhinoceros inside PAs and reduce the occurrence of crop-raiding in farmlands. This includes maintaining the environmental flows required to support high-quality grasslands, as mentioned above for tiger prey species. In addition, electric fences must be well maintained so that they continue to be effective.

Bears and other species (wild water buffalo, wild boar, and gaur) were less pronounced conflict species. Wild water buffalos in particular survive in an isolated and small reserve (Koshi Tappu Wildlife Reserve) in eastern Nepal where they frequently attack people. Plans are underway to translocate some of these animals to Chitwan National Park, which is unoccupied by people and contains high-quality habitat that is within the former geographic range of wild water buffalos. We suggest that these plans should include strategies to reduce human-buffalo conflict, as suggested by Heinen and Paudel [58]. Attacks by wild boar are not common, although this species poses a serious problem as a crop raider [26].

Our study demonstrates that human-dominated landscapes and not Pas are the major wildlife conflict hotspots in Nepal. The majority of these conflicts involved leopards and elephants, and people were more likely to be killed in their homesteads by these wildlife species (Table 2) than by other species. There was a decrease in conflict events in 2014 for elephants and leopards (Fig 4). Such a sharp decrease may be a combined result of technical measures used to mitigate human–wildlife conflict (e.g., electric fences and predator-proof corrals) and increased public awareness about animal behavior (e.g., avoiding making noises or engaging in other behaviors, such as human movement at night, that might provoke wildlife aggression). We emphasize that technical measures may not be the sole explanation for these reductions because (a) electric fences are confined mainly within the jurisdictions of parks and have not been effective due their poor quality (e.g., inadequate poles and wires), lack of a regular power supply and maintenance and the socio-economic conditions at the local level (e.g., people remove fences to allow free movement of their livestock into forests (park managers, pers. comm.). High winter rain levels in 2014 (50% above normal) compared with the previous four years [59], for example, may have also contributed to the avoidance of potential encounters by (a) providing wildlife with food/water in the forests and (b) limiting human activities within their villages. We argue that further research based on long-term data is necessary to ascertain whether such fluctuations are attributable to these factors.

Most victims (a) are frequent forest visitors, collecting firewood or fodder or grazing their livestock; (b) reside in small, poorly secured mud houses located adjacent to or near forests along with belongings that might attract wildlife (e.g., livestock, food-grain); and (c) attempt to chase off wildlife using rudimentary tools (e.g., locally made sound boxes and burning sticks). Thus, any conflict mitigation plan should focus on the socio-economic issues of local populations and the ecology of the wildlife involved to create non-overlapping resources for both groups [3,60,61].

## Conclusions, Conservation Implications and Future Research

Nepal has eliminated the poaching of rhinoceros since 2011 (also known as zero poaching) [62]. Some reports even suggest that increases in the tiger and rhinoceros populations are occurring [47,63], and community forestry has been successful in restoring locally extirpated wildlife populations. However, these accomplishments may have been achieved at the cost of an increasing number of wildlife conflicts occurring outside PAs.

Our results suggest that elephants and leopards should be the main focus of management efforts to minimize injury and the loss of human life and mitigate human-wildlife conflicts. This is based on three major findings: attacks by these species were (a) the most frequent, (b) common outside Pas (spatial dimension), and (c) associated with a high human fatality rate. Earlier attempts to resolve conflicts were confined mainly within the jurisdiction of PAs and included, among other strategies,(1) the deployment of electric fences to prevent wildlife movement towards human settlements, (2) building predator-proof corrals to prevent livestock loss by predators at night, and (3) the planting of crops that are unpalatable to wildlife, such as peppermint. These mitigation strategies undoubtedly helped to reduce conflict. However, the efficacy of such measures at a national level is low because there is minimal infrastructure in places where it is urgently needed to address some of these issues. The widespread common leopard, for example, causes conservation conflicts along the entire mid-hill region of Nepal, far from PAs, but district forest offices have no institutional capacity to respond (e.g., capturing leopards, engaging in conservation planning and monitoring animals). The same is true for dealing with conflicts with elephants in lowland Nepal. Therefore, there is an urgent need to build the institutional capacity to address conflicts with these two species as part of the framework of overall conservation planning [3,61]. Here, we provide species-specific recommendations to guide future research and conservation activities in Nepal with the goal of reducing human-wildlife conflict (Table 3).

**Table 3:** Ecological and conflict issues and management recommendations.

Wildlife	Ecological and conflict issues	Management recoqwmmendations
Elephant	—High frequency of attacks, with an extremely high kill ratio (67%)	—Restore corridors in critical areas along elephant migratory routes
	—Attacks occurred mostly in human-dominated landscapes (farmland and homes)	—Prepare a well-planned preventive mechanism (e.g., early warning system)
	—Attacks peaked in December	—Educate and train local residents about animal behavior
		—Protect villages with electric fences
Leopard	—Rapidly increasing rate of attacks	—Develop a network of community-based protected areas in the mid-hills and lower mountains
	—Almost all attacks occurred outside protected areas	
	—Attacks peaked in the dry months	—Incorporate wildlife management and conservation practices in community forestry programs (e.g., leopard-proof corrals)
		—Educate and train local residents about animal behavior
Tiger	—Attacks occurred mostly within protected areas and forests	—Maintain healthy prey populations
		—Maintain environmentally sustainable flows in critical rivers to maintain prey habitats
		—Reduce human dependence on forest resources
		—Identify and designate critical tiger habitats in protected areas and conservation landscapes, and prohibit human movement in such areas
Rhinoceros	—Attacks peaked in the dry season	—Restore grasslands and oxbow lakes to restore habitat in protected areas. Maintain these areas to ensure continued environmentally sustainable flows in critical rivers
	—Attacks occurred within protected areas	
		—Maintain and expand electrified fences to protect farmlands
		—Reduce human dependence on forest resources
		—Educate and train local residents about animal behavior

This study focused only on human injury and death; it did not look at the loss of livestock, crops and other human property. We recommend that future studies be conducted examining these aspects, which are likely to result in further recommendations for human-wildlife conflict mitigation.

## Supporting Information

S1 File Data collection strategies. (DOCX)

S2 File Descriptive statistics of variables. (DOCX)
