# Supplementary material for: Human-Wildlife Conflicts in Nepal: Patterns of Human Fatalities and Injuries Caused by Large Mammals
Source: PLoS One. 2016 Sep 9;11(9):e0161717. doi: 10.1371/journal.pone.0161717 (PMC5017643; doi:10.1371/journal.pone.0161717)
Supplement: S1 File — (DOCX) [file pone.0161717.s001.docx]

**S1 File. Data collection strategies**

Data on human injuries and fatalities caused by wildlife were collected from the Ministry of Forests and Soil Conservation (MoFSC), Nepal. The MoFSC is solely responsible for carrying out programs related to sustainable forest management and biodiversity conservation in Nepal. The MoFSC formulated guidelines for the distribution of monetary relief to the victims of wildlife-related losses on January 31, 2006 (first amendment on May 14, 2015). According to the guidelines, each of the five Regional Forest Directorates (RFDs) (Figure 1c) is responsible for providing monetary relief to wildlife-related losses in its respective jurisdiction. RFDs receive recommendations for monetary relief disbursement from either the District Forest Offices (DFOs) or Protected Area Offices (PAOs) (See Figure S1 for a flow diagram of the procedure). There is a well-defined jurisdictional boundary between these two government agencies. If the incident occurs inside protected areas and/or their buffer zones, formal procedures are conducted through the respective PAO. Buffer zones are designated areas surrounding protected areas that may include forests, human settlements and agriculture lands. For all other incidents (outside of PAs), DFOs are responsible for processing applications for relief claims made by victims.

District Forest Office (Fig. 1)

(75 DFOs in Nepal)

An incident occurs (attacks by wildlife)

Respective DFO or PAO evaluates application and makes recommendation to RFD

Victim(s) and/or their families submit applications

Protected Area Office (PAO) (Fig. 1)

(20 PA offices)

Regional Forest Directorate (RFD)

(5 RFDs in Nepal, Fig. 1)

RFD releases funds

RFD releases funds

RFD releases funds

PAO disburses monetary relief

DFO disburses monetary relief

Figure S1: A schematic of the procedure for disbursing financial relief to the victims of wildlife attacks in Nepal

In the guidelines, a rigorous verification protocol has been outlined to minimize false claims. For example, a person cannot make a claim for crop damage by a tiger. The guidelines require multiple lines of evidence to make a claim based on the types of damage. The required evidence could be (a) a photograph of the victim (if applicable), (b) death certificate in cases of human fatality (if applicable), (c) public inquiry into the incident, (d) report from a hospital (postmortem report if applicable), (e) police report, (f) official letter from a relevant organization supporting the claims (e.g., buffer zone community forest, conservation area management council, community forest user group), (g) letter of recommendation from a respective village development committee and municipality, and (h) reports from an agriculture technician or a veterinarian (if applicable). Once all requirements are met, the application is forwarded to a recommendation committee formed at a DFO or PAO. If the committee finds the claim to be valid based on the veracity of the documents, the application is then forwarded to the RFD for disbursement. The guidelines require the RFD to submit a quarterly report to the MoFSC including a survey report based on a sampling of at least 10 % of the population of recipients.

Data on relief distributions have been exhaustively collected from the official records at RFDs. First, RFDs were visited, and official archives were searched for documented cases of human-wildlife conflict. During such visits, the staff was consulted to ensure a thorough review of all archives. Second, data were compiled in a spreadsheet and cross-checked for any inconsistencies (e.g., unreadable text in the old archive and missing information). In cases where there was any doubt, staff in the respective DFO and PAO were consulted. It was found that not all conflict cases reported due to a lack of information among the people and the lengthy administrative process required to submit an application. However, it was noted that almost all conflict cases involving the death and/or injury of humans were reported and documented at RFDs. We, therefore, strictly adhered to the documented database because (a) conflict species were identified correctly in the verification process (with the involvement of forest staff and local people), (b) the time and location of incidents were clearly specified in the public enquiry report and (c) cases of false claims were highly unlikely (please see the Methods for detailed information).
