# Supplementary material for: Human-Wildlife Conflicts in Nepal: Patterns of Human Fatalities and Injuries Caused by Large Mammals
Source: PLoS One. 2016 Sep 9;11(9):e0161717. doi: 10.1371/journal.pone.0161717 (PMC5017643; doi:10.1371/journal.pone.0161717)
Supplement: S2 File — (DOCX) [file pone.0161717.s002.docx]

**S2 File. Descriptive statistics of variables.**

Descriptive statistics (number of categories, mode, mode frequency and relative frequency per categories in %) of different variables (month, condition, season, wildlife, inside or Outside PA and human fatality and injury), based on nationwide survey of human death and injury by attacks of large mammals in Nepal

| Variables | No. of categories | Mode | Mode frequency | Category | Rel. frequency per category (%) |
| --- | --- | --- | --- | --- | --- |
| Month | 12 | Dec | 77 | Apr | 7.13 |
|  |  |  |  | Aug | 6.70 |
|  |  |  |  | Dec | 16.63 |
|  |  |  |  | Feb | 7.13 |
|  |  |  |  | Jan | 13.39 |
|  |  |  |  | Jul | 9.07 |
|  |  |  |  | Jun | 4.54 |
|  |  |  |  | Mar | 4.97 |
|  |  |  |  | May | 7.99 |
|  |  |  |  | Nov | 6.48 |
|  |  |  |  | Oct | 10.15 |
|  |  |  |  | Sep | 5.83 |
| Condition | 3 | Forest | 201 | Farmland | 33.91 |
|  |  |  |  | Forest | 43.41 |
|  |  |  |  | Home | 22.68 |
| Season | 4 | Winter | 172 | Autumn | 22.46 |
|  |  |  |  | Spring | 20.09 |
|  |  |  |  | Summer | 20.30 |
|  |  |  |  | Winter | 37.15 |
| Wildlife | 6 | Elephant | 137 | Bear | 11.88 |
|  |  |  |  | Elephant | 29.59 |
|  |  |  |  | Leopard | 20.95 |
|  |  |  |  | Other | 9.72 |
|  |  |  |  | Rhinoceros | 18.36 |
|  |  |  |  | Tiger | 9.50 |
| Inside or Outside PA | 2 | Outside PA | 237 | Outside PA | 51.19 |
|  |  |  |  | Inside PA | 48.81 |
| Events | 2 | Injury | 286 | Injury | 61.77 |
|  |  |  |  | Fatality | 38.23 |
